# Melatonin effects on the left ventricular function in neonates with persistent pulmonary hypertension

**DOI:** 10.1007/s00431-026-06864-z

**Published:** 2026-04-09

**Authors:** Sarah M. Nofal, Abdel-Rahman M. El-Mashad, Asmaa M. Elmesiry, Amany M. Abouelenain, Mostafa M. Awny

**Affiliations:** 1 Pediatric and Neonatology Department, Faculty of Medicine, Tanta University, El Bahr St., Tanta Qism 2, First Tanta, Gharbia Governorate, Tanta, 31111 Egypt; 2 Clinical Pathology Department, Faculty of Medicine, Tanta University, Tanta, Egypt

**Keywords:** Persistent pulmonary hypertension, Melatonin, Left ventricular function, Advanced echocardiography, Cardiac biomarkers

## Abstract

The aim of this study is to investigate the effects of melatonin on the left ventricular (LV) function in persistent pulmonary hypertension of the newborn (PPHN) and to assess its potential role in enhancing cardiac performance and reducing oxidative stress. This randomized controlled trial was conducted on 80 neonates (≥ 36 weeks’ gestational age) with PPHN confirmed by echocardiography (Echo). Participants were assigned to receive standard therapy alone or in combination with oral melatonin. LV function was evaluated using echocardiographic parameters, and levels of serum biomarkers of oxidative stress and inflammation were also measured. Unlike the placebo group, the melatonin group exhibited significantly better LV function, regarding the conventional and advanced echocardiographic parameters. Additionally, melatonin administration was associated with earlier weaning from mechanical ventilation (MV) and respiratory support, shorter hospital stays, lower levels of serum high-mobility group box-1 (HMGB1) protein, and N-terminal pro-B-type natriuretic peptide (NT-proBNP).

*Conclusion*: Melatonin can enhance LV function in PPHN, likely through its cardioprotective and antioxidative mechanisms. These findings support melatonin as a promising adjunctive therapy in neonatal pulmonary hypertension, warranting further investigation in larger clinical trials.

*Trial registration*: registered on ClinicalTrials.gov (ID: NCT07090720), URL: https://clinicaltrials.gov/study/NCT07090720?cond=NCT07090720&rank=1, Date: (29/7/2025).

**Table:** 

**What is Known:** • *PPHN induces hypoxic respiratory failure, jeopardizing multiorgan function, including LV performance*. • *Oxidative stress is crucial in pulmonary arterial hypertension development*.	
**What is New:** • *Melatonin’s antioxidant and vasodilatory properties may serve as an adjunctive therapy in PPHN and enhance myocardial protection from the hypoxic effect of PPHN*.

**Supplementary Information:**

The online version contains supplementary material available at 10.1007/s00431-026-06864-z.

## Introduction

Persistent pulmonary hypertension of the newborn (PPHN) represents a life-threatening failure of transitioning from the fetal into the postnatal circulation [1]. Its incidence is approximately 1 in 500–1000 live births, with a mortality of 3.0–57.9% [2].

The sustained increase in pulmonary vascular resistance (PVR) diminishes pulmonary blood flow, exacerbates systemic and cerebral hypoxia, and leads to systemic hypotension through peripheral vasodilation, compromised left ventricular (LV) function, and reduced preload [3].

Neonatologist-performed echocardiography (NPE) supports diagnosis, grading, therapy selection, and treatment monitoring, with published guidance for pulmonary hypertension management [4]. Conventional indices, including ejection fraction and shortening fraction, exhibit reduced sensitivity to subtle dysfunction compared to advanced modalities [5].

Melatonin, an antioxidant not produced by infants during early life, may improve endothelial function and pulmonary vasodilation, potentially through the activation of prostanoid pathways in PPHN [6].

High-mobility group box-1 (HMGB1) protein, an inflammatory marker, may serve as an early biomarker in hypoxia-provoked PPHN [7]. Natriuretic peptides (B-type natriuretic peptide (BNP) and N-terminal pro-B-type natriuretic peptide (NT-pro-BNP)) can indicate myocardial injury and ventricular load, potentially assisting in the evaluation of severity and treatment response. Both are significant in the pathophysiology of PPHN, with their serum concentrations serving as potential biomarkers for predicting treatment response and assessing disease severity [8].

This work aimed to assess the potential protective effect of melatonin therapy on LV function following PPHN-related hypoxia using conventional and advanced echocardiography, and correlate echocardiography measures with serum HMGB1 and NT-pro-BNP in neonates ≥ 36 weeks diagnosed with PPHN.

## Patients and methods

### Study design and setting

This randomized controlled trial enrolled 80 neonates ≥ 36 weeks’ gestation, both sexes, who met the diagnostic criteria of PPHN [9, 10]. This trial was conducted at the Neonatal Intensive Care Unit (NICU), Tanta University Hospitals, Egypt, between January 2023 and January 2025.

The trial was approved by the Ethical Committee of the Faculty of Medicine, Tanta University (approval number: 36173/12/22) and registered at ClinicalTrials.gov (Identifier: NCT07090720; registration date: 29 July 2025). The study was conducted in accordance with the Declaration of Helsinki and the principles of Good Clinical Practice. Written informed consent was obtained from the caregivers of all enrolled neonates prior to participation.

Inclusion criteria were neonates with a gestational age of ≥ 36 weeks who fulfilled the diagnostic criteria for PPHN.

Exclusion criteria included preterm birth < 36 weeks’ gestation, congenital heart disease (including significant shunts), clinically significant arrhythmias, chromosomal abnormalities, surgical conditions, severe pathological jaundice, metabolic disorders, major congenital malformations, acute renal injury, and severe perinatal asphyxia (Apgar score < 3 at the 5th minute).

### Randomization and blinding

Randomization and allocation concealment were executed utilizing a computer-generated software (randomizer.org) with a 1:1 ratio; assignments were disguised within sequentially numbered, opaque, sealed envelopes to maintain blinding. Randomization was performed immediately after confirmation of eligibility and completion of baseline clinical and echocardiographic assessment. Allocation was revealed only after enrollment, and the assigned intervention was initiated within the same admission period following randomization.

Due to the nature of the intervention, treating clinicians were aware of group allocation; however, outcome assessors (echocardiography analysts and laboratory personnel) were blinded to allocation.

Eighty neonates diagnosed as PPHN were randomized: Melatonin group (*n* = 40) received standard care with melatonin (10 mg/kg/day) for five doses started within the first 24 h; melatonin tablets (Puritan’s Pride, Oakdale, NY, USA) were crushed, suspended in 5 mL normal saline, and given by an orogastric tube. The dose was selected based on prior neonatal experience demonstrating safety at comparable regimens [11, 12]. The control group (*n* = 40) received standard care in addition to placebo administered via an orogastric tube, using the same volume and dosing schedule as the melatonin group.

### Clinical and laboratory assessment

All the enrolled neonates underwent full medical history taking, clinical examination, and laboratory testing. They included complete blood count (CBC), liver and kidney function test, and electrolytes and arterialized or capillary blood gases (ABG\CBG).

### Research biomarker assays

For research analysis, venous blood was drawn on admission and after completion of melatonin course. Serum was separated, aliquoted, and maintained at − 20 °C (or − 80 °C), and repeated freeze–thaw cycles were avoided. Serum HMGB1 and NT-pro-BNP were quantified using enzyme-linked immunosorbent assays (ELISA; Wuhan Huamei Biotech) per the protocols. Absorbance was read at 450 nm, and concentrations were interpolated from standard curves. All serum samples were analyzed in duplicate, and the mean of duplicate readings was used for statistical analysis. According to the manufacturer’s datasheets (Wuhan Huamei Biotech, China), the analytical performance of the ELISA kits demonstrated acceptable reliability, with intra-assay coefficients of variation (CVs) < 10% and inter-assay CVs < 12% for both HMGB1 and NT-proBNP assays. All assays were performed according to the manufacturer’s instructions, and samples from the two study groups were analyzed within the same assay runs to minimize inter-assay variability. Blood gas within first hour.

### Echocardiographic assessment

- Equipment and general protocol

A comprehensive transthoracic Echo was applied on Vivid 7 or 9, GE Healthcare, Horten, Norway, platforms with offline analysis utilizing Echo PAC software (Echo PAC PC, 204; GE, Horten, Norway), following methods aligned with the American Society of Echocardiography [13].

The echocardiography was performed by a qualified neonatologist, and the offline analysis was conducted by a trained research analyst. Both the echocardiography and the offline analysis were carried out by individuals who were masked to randomization.

- Timing of echocardiographic evaluations

A baseline 2D echocardiography was initially done on admission, which included a full structural and functional assessment. Data related to PPHN and LV function were repeated 24 h following the end of therapy (EOT) together with TDI and 2D/3D STE.

- Conventional functional echocardiography

The initial evaluation of conventional parameters included diagnosis of PPHN, including estimated systolic pulmonary arterial pressure (ESPAP), main pulmonary artery (MPA) diameter, tricuspid annular plane systolic excursion (TAPSE), and fractional area change (FAC) measurements routinely included as part of our protocol for assessing right ventricular systolic function.

LV systolic function was assessed by the LV fractional shortening (LVFS) by motion mode (M-mode), and LV ejection fraction (LVEF) by the Simpson biplane method.

- Tissue Doppler imaging

Pulsed-wave tissue Doppler imaging at the lateral mitral annulus was deployed to measure peak systolic (s′), early (e′), and late diastolic (a′) velocities. Additionally, LV myocardial performance index (MPI) was derived from time intervals, specifically, isovolumic contraction/relaxation time (IVCT)/(IVRT), and ejection time (ET), using the formula: MPI = (IVCT + IVRT)/ET [14].

Two-dimensional speckle tracking echocardiography

High-frame-rate grayscale images from apical four-, two-, and three-chamber views at high frame rates (≥ 60 fps) were analyzed to compute global longitudinal strain (GLS) using a 17-segment LV model, with manual endocardial tracing and verification of tracking quality [15].

Circumferential strain was ascertained in the short-axis view, acquired from the standard parasternal window at the basal level and from an anterior or anterolateral position at the apical level. To ensure standardized image acquisition, the basal plane was defined as the level intersecting the tips of the mitral valve leaflets. In contrast, the apical plane was positioned immediately proximal to the point of end-systolic LV cavity obliteration and distal to the papillary muscles. Particular care was taken to achieve a circular LV cross-sectional geometry to optimize strain measurement accuracy [16].

- Three-dimensional echocardiography

Multi-beat full-volume datasets (stitched over 4–7 cardiac cycles) were analyzed with 4D Auto LV quantification to obtain three-dimensional end-diastolic and end-systolic volumes, ejection fraction, fractional shortening, and a 3D LV model.

### Outcomes

The primary outcome was LV function at EOT, with LV-GLS as the principal endpoint. Additional echocardiographic indices, including LVEF, LVFS, TDI-derived MPI, and 2D/3D-STE parameters, were considered supportive components of the primary outcome.

Secondary outcomes included serum HMGB1 and NT-pro-BNP levels measured at EOT and their correlations with echocardiographic parameters. Additional secondary clinical outcomes were duration of MV, duration of non-invasive respiratory support, and NICU length of stay.

### Sample size calculation

Sample size was calculated using G*Power (University of Kiel, Germany). A minimum of 40 participants/group was determined to provide 95% statistical power to detect a 15% relative improvement in LV-GLS, the primary outcome, following melatonin administration, assuming a two-tailed α error of 0.05. This calculation was based on prior data [17] reporting a mean LV-GLS of − 16.7% ± 3.3% in patients with PPHN.

### Statistical analysis

Statistical analyses were performed through IBM SPSS Statistics (v20.0; IBM Corp., Armonk, NY). Categorical variables are reported as frequencies and percentages, while continuous variables are reported as mean ± standard deviation or median with IQR, depending on distribution; ranges were provided for descriptive context. Normality was assessed using the Shapiro–Wilk test. Between-group comparisons of normally distributed continuous variables employed the independent Student’s *t*-test; non-normally distributed variables were analyzed using the Mann–Whitney *U* test. Categorical variables were compared using Pearson’s chi-square test, with a Monte Carlo simulation applied when > 20% of expected cell counts were < 5. Correlations were evaluated using Spearman’s rank correlation coefficient. The ROC curve analysis was conducted to assess the diagnostic performance of the variables used to detect cardiac involvement. Intra-observer variability was assessed by repeating measurements in ten randomly selected neonates after a 2-month interval; inter-observer variability was determined by a second, blind observer independently analyzing the same subset. Statistical significance was defined as *p* < 0.05.

## Results

**Fig. 1:**
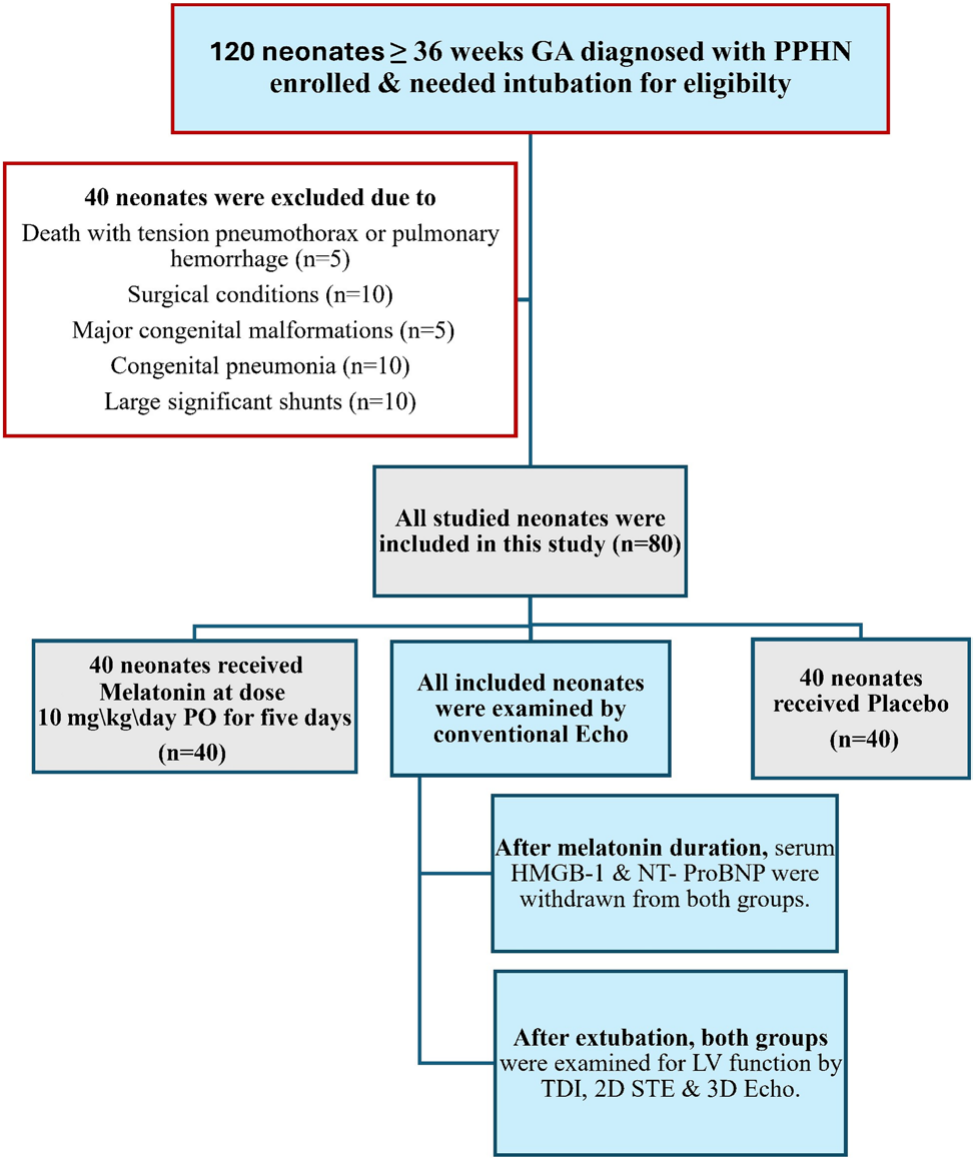
Study flowchart

Figure 1 depicts the study flowchart.

Table 1 demonstrates no significant difference in (sex, gestational age, postnatal age, weight of birth, mode of delivery, main respiratory disorders triggering PPHN, and Vasoactive-inotropic score) between groups and indicates significantly lower APGAR scores at 5th min, SNAPPE-II score in melatonin group than control group (*p* < 0.05).

**Table 1:** Demographic data

			Melatonin group (*n* = 40)	Control group (*n* = 40)	***P***
Sex	Male		26 (65%)	28 (70%)	0.633
	Female		14 (35%)	12 (30%)	
Gestational age (weeks)			38.03 ± 1.17	38.40 ± 1.28	0.174
Postnatal Age (days)			1.60 ± 0.63	1.90 ± 0.78	0.083
Weight of birth (kg)			3.12 ± 0.33	3.18 ± 0.28	0.339
APGAR 5 min			5.15 ± 0.89	6.55 ± 0.93	** < 0.001**
SNAPPE-II score			39.15 ± 3.86	40.83 ± 2.82	**0.030**
Mode of delivery		Normal vaginal delivery	7 (17.5%)	8 (20%)	0.775
		Cesarean section	33 (82.5%)	32 (80%)	
Main respiratory disorders triggering PPHN		Meconium aspiration syndrome	17 (42.5%)	15 (37.5%)	0.648
		Congenital pneumonia	16 (40%)	14 (35%)	0.485
		Respiratory distress syndrome	7 (17.5%)	5 (12.5%)	0.531
		Early onset sepsis (EOS)	11 (27.5%)	9 (22.5%)	0.606
Vasoactive-inotropic score			15.35 ± 3.21	16.78 ± 4.74	0.120

Data interpretation: mean ± standard deviation or frequency (%)

Table 2 indicates significantly higher serum urea and creatinine levels in melatonin group (*p* < 0.001) before the start of therapy, with no significant differences in Downe’s score, vital signs, CBC, and ABG\CBG, liver and kidney function test, and electrolytes. Also, no significant differences were observed on admission as regards the conventional echocardiography parameters, oxygenation index, mean airway pressure, oxygenation saturation index, and SpO_2_/FiO_2_.

**Table 2:** Clinical and laboratory metrics on admission

	Melatonin group (*n* = 40)	Control group (*n* = 40)	***P***
Downe’s score	8.23 ± 0.66	8.20 ± 0.69	0.869
Heart rate (bpm)	133.6 ± 12.80	133.6 ± 12.80	1.000
Systolic blood pressure (mmHg)	77.70 ± 3.87	77.70 ± 3.87	1.000
Mean blood pressure (mmHg)	43.58 ± 3.35	44.90 ± 2.92	0.063
Oxygenation index (cmH2O)	17.4 ± 4.14	15.98 ± 3.48	0.099
Peak airway pressure (cmH2O)	16.68 ± 4.78	15.45 ± 3.25	0.184
Oxygenation saturation index	8.23 ± 2.09	7.93 ± 1.62	0.476
SpO2/FiO2 ratio	264.35 ± 29.46	276.18 ± 28.82	0.073
Hemoglobin (g/dL)	15.24 ± 1.26	15.61 ± 1.47	0.225
Total leukocyte count (× 10⁹/L)	11.08 ± 2.21	11.57 ± 1.88	0.287
Platelets (× 10⁹/L)	357.0 ± 96.28	357.0 ± 96.28	1.000
pH	7.28 ± 0.11	7.28 ± 0.11	1.000
Carbon dioxide partial pressure (mmHg)	50.08 ± 12.64	49.70 ± 12.56	0.827
Oxygen partial pressure (mmHg)	60.8 ± 6.32	61.05 ± 6.18	0.858
Base deficit (mmol/L)	5.02 ± 2.11	5.13 ± 1.96	0.81
Urea (mg/dL)	55.43 ± 17.27	36.14 ± 8.99	** < 0.001**
Creatinine (mg/dL)	0.81 ± 0.28	0.41 ± 0.16	** < 0.001**
Alanine aminotransferase (ALT) (U/L)	31.70 ± 6.43	31.70 ± 6.43	1.00
Aspartate aminotransferase (AST) (U/L)	30.63 ± 6.24	30.63 ± 6.24	1.00
Albumin (g/dL)	3.14 ± 0.19	3.14 ± 0.19	1.00
Serum sodium (mEq/L)	139.3 ± 6.63	139.3 ± 6.63	1.00
Serum potassium (mEq/L)	4.46 ± 0.94	4.46 ± 0.94	1.00
Serum Ionized calcium (mmol/L)	1.03 ± 0.16	1.02 ± 0.17	0.432

Data interpretation: mean ± standard deviation or frequency (%)

Table 3 shows EOT echocardiography with better LV systolic function, reduced ESPAP, better 2D\3D strain values, and lower MPI in melatonin group.

**Table 3:** Echocardiographic measurements

		Melatonin group (*n* = 40)	Control group (*n* = 40)	*P*
Conventional echo at diagnosis of PPHN	ESPAP_1_	74.25 ± 6.89	70.25 ± 10.12	0.043
	MPA_1_	11.87 ± 3.28	12.32 ± 3.04	0.534
	TAPSE_1_	8.53 ± 0.84	8.41 ± 0.77	0.488
	FAC_1_ (%)	33.26 ± 3.56	33.04 ± 2.97	0.765
	LVEF₁ (%)	65.47 ± 2.44	64.58 ± 2.33	0.099
	LVFS₁ (%)	34.70 ± 1.27	34.07 ± 1.98	0.094
Conventional Echo follow-up after EOT	ESPAP_2_	43.60 ± 4.30	50.50 ± 4.91	< 0.001
	MPA_2_	7.72 ± 0.26	9.49 ± 1.22	< 0.001
	TAPSE_2_	9.41 ± 0.24	8.68 ± 0.52	< 0.001
	FAC_2_ (%)	35.98 ± 4.63	35.73 ± 4.40	0.846
	LVEF₂ (%)	67.65 ± 0.63	64.87 ± 1.55	< 0.001
	LVFS₂ (%)	36.89 ± 1.36	34.68 ± 1.27	< 0.001
TDI after EOT	LV free wall e′/a′	1.00 ± 0.08	0.92 ± 0.14	0.003
	e′ (cm/s)	5.99 ± 0.32	5.36 ± 0.81	< 0.001
	a′ (cm/s)	6.06 ± 0.49	5.70 ± 0.63	0.006
	s′ (cm/s)	6.83 ± 0.50	5.63 ± 0.82	< 0.001
	LV MPI	0.41 ± 0.03	0.47 ± 0.06	< 0.001
2D STE after EOT	LV GLS (%)	− 21.84 ± 1.62	− 18.83 ± 1.31	< 0.001
	LV GCS (%)	− 19.98 ± 1.46	− 18.49 ± 1.96	< 0.001
3D Echo after EOT	LVEF (%)	65.29 ± 1.54	64.34 ± 1.18	0.003
	LVFS (%)	33.98 ± 1.03	33.05 ± 0.99	< 0.001
	LV GLS (%)	− 22.29 ± 1.70	− 20.16 ± 1.60	< 0.001
	LV GCS (%)	− 32.78 ± 0.81	− 31.54 ± 0.74	< 0.001

Table 4 shows the baseline HMGB-1 and NT-pro-BNP which show no significant difference between two groups. However, melatonin group demonstrated significantly lower serum HMGB-1 and NT-pro-BNP concentrations along with shorter duration of MV and less need for oxygen therapy and shorter length of NICU stay compared to control group.

**Table 4:** Serum biomarkers and the secondary outcomes after EOT

			Melatonin group (*n* = 40)	Control group (*n* = 40)	*P*
Serum biomarkers	HMGB-1 (pg/mL)	Baseline	3950.88 ± 1454.14	4227.77 ± 1517.37	0.407
		After EOT	3082.3 ± 606.1	4973.4 ± 900.8	< 0.001
	NT-proBNP (pg/mL)	Baseline	3473.92 ± 1500.95	3691.54 ± 1580.9	0.530
		After EOT	1181.5 ± 166.3	2207.6 ± 695.5	< 0.001
MV duration (days)			7.28 ± 0.75	11.45 ± 1.75	< 0.001
Oxygen duration (days)			9.43 ± 0.64	15.80 ± 1.73	< 0.001
NICU stay length (days)			11.95 ± 1.04	18.53 ± 1.93	< 0.001

Data interpretation: mean ± standard deviation or frequency (%)

*HMGB-1* high-mobility group box-1, *NT-proBNP* N-terminal pro B-type natriuretic peptide, *MV* mechanical ventilation

Serum HMGB-1 concentrations showed significant negative correlations with late diastolic a′ velocity, 2D STE parameters, 3D LVEF, 3D LV-GLS, and 3D LV-GCS, and a significant positive relation to LV MPI after EOT in either group (Table 1 Supplementary file).

Table 2 Supplementary file illustrates that, melatonin group, serum NT-pro-BNP exhibited a substantial positive association with LV MPI and significant negative relationships with 2D LV GLS, 2D LV GCS, 3D LVEF, and 3D LV GLS after EOT in either group.

## Discussion

Failure of transition may cause hypoxemic respiratory failure (HRF) and PPHN [18]. Functional neonatal echocardiography is a crucial instrument for verifying the diagnosis of PPHN, selecting targeted therapy, and evaluating treatment responses [19].

Pulmonary hypertension invariably results in right ventricular dysfunction, as the increased pressure burden reduces both systolic and diastolic ventricular velocities. The LV may be influenced by a common underlying pathology associated with PPHN, detrimental interactions between the ventricles, or extensive secondary effects related to PPHN, including hypoxia, acidosis, dependence on MV, and administration of inotropic agents [3].

Melatonin provides significant protection against reactive oxygen species (ROS). This protection relies on three established properties: direct ROS scavenging, activating antioxidant enzymes, and inhibiting ROS production in pro-oxidant agents [20]. To our knowledge, no prior research exists regarding the cardioprotective benefits of melatonin on human newborns diagnosed with PPHN.

Our study showed significantly shorter durations of invasive MV, oxygen support, and length of NICU stay in melatonin group, who received a 5-day course of melatonin along with standard PPHN therapy, than in controls, who only received the standard therapy alone.

Oxidative stress is a key contributor to pulmonary arterial hypertension (PAH) pathogenesis, primarily by reducing endothelium-derived relaxing factors and diminishing nitric oxide bioavailability. In response, antioxidant-based therapeutic strategies have been explored in perinatal medicine, with melatonin emerging as a particularly advantageous agent due to its favorable pharmacological profile. Furthermore, melatonin’s antioxidant efficacy has been substantiated in neonatal pulmonary and respiratory disorders, demonstrating therapeutic relevance in this vulnerable population [6].

Previous clinical trials evaluating melatonin as a supplement to therapeutic hypothermia in hypoxic-ischemic encephalopathy (HIE) neonates demonstrated the neuroprotective and systemic benefits of melatonin, suggesting its potential utility as an adjunctive therapy in hypoxia associated with PPHN [11, 21, 22].

Our findings align with those of Figueroa et al. [6], who first demonstrated that oral administration of melatonin (1 mg/kg) during early postnatal life in neonatal sheep exposed to hypoxia modulates the expression of vasoactive prostanoid pathways within the hypertensive pulmonary vasculature. Specifically, melatonin altered the balance of both vasodilatory and vasoconstrictive prostanoid signalling, suggesting a regulatory role in pulmonary vascular tone under hypoxic conditions.

Our study showed that serum HMGB-1 and NT-pro-BNP levels were significantly lower in melatonin group relative to controls, which can additionally predict the beneficial effects of melatonin in PPHN\HRF.

NT-pro-BNP is a non-intrusive metric for assessing PPHN. It was determined that there was a strong link with echocardiography indices. In comparison to echocardiography, NT-pro-BNP is readily accessible, making it a viable choice for PPHN screening [23]. This aligns with Tüfekcı et al. [24], who found markedly heightened NT-pro-BNP levels in neonates with PPHN relative to healthy newborns.

Serum HMGB1 levels were considerably higher in newborns with PPHN, and dropped markedly following PPHN resolution, indicating that HMGB1 is associated with the pathophysiology of hypoxia-induced PPHN. Alterations in HMGB1 may serve as an early diagnostic marker for hypoxia-induced PPHN [25].

Our study revealed no significant difference between groups in conventional echocardiography measurements at the time of PPHN diagnosis, except that ESPAP was considerably elevated in melatonin group in contrast to controls. After EOT, ESPAP became lower in melatonin group, which can prove the advantageous effects of melatonin in PPHN. The MPA EOT in melatonin group was substantially less. Meanwhile, LVEF and LVFS were significantly higher in melatonin group than in controls after EOT.

This can be compared to Abdelmassih et al. [26], who conducted a prospective case–control study including 30 full-term infants with PPHN and 30 healthy babies, and revealed no significant differences in LVEF and LVSF between the two groups. On the other hand, Le Duc et al. [27] showcased no significant difference in LVSF between the severe PPHN group (mean = 50.19% ± 14.87%) and the asymptomatic PH group (mean = 48.82% ± 11.68%) (*p* = 0.55).

Our study showed that TDI parameters showed that LV free wall e′/a′ ratio, e′, a′, and s′ wave velocities were significantly higher in melatonin group. Meanwhile, LV MPI was significantly higher in controls, meaning that TDI parameters showed better results in melatonin group after EOT.

Tissue Doppler indicators elucidate disparities between newborns in normal and diseased states that traditional indices fail to identify, as they are less influenced by loading conditions [28].

Our study additionally showed that the 2D STE parameters after EOT (2D LV GLS and 2D LV GCS) were significantly higher in melatonin group. This aligns with Baz et al. [29], who determined that LV global strain is a crucial predictor of the LV performance in patients with hemodynamically significant patent ductus arteriosus, surpassing other traditional Echo parameters and TDI.

3D Echo parameters, in our study, included 3D LVEF, 3D LVFS, 3D LV GLS, and 3D LV GCS; all measurements were higher in melatonin group, after EOT. Automated 3D LV quantification, echocardiography, and 3D speckle tracking are dependable instruments for evaluating LV volume and systolic function [30]. To our knowledge, no prior investigations have assessed 3D STE parameters of the LV in PPHN.

Our study demonstrated a negative link between serum HMGB-1 concentration post-EOT and LV late diastolic wave a′ velocity in melatonin group, alongside a positive correlation between serum HMGB-1 levels, NT-pro-BNP levels, and LV MPI, indicating that both indicators are indicative of LV function. The MPI value exhibits an inverse correlation with myocardial function; an increase in MPI signifies a decline in global myocardial function [29].

Our study showed a negative correlation between serum HMGB-1 and 2D and 3D Echo parameters in melatonin group, which means that the HMGB-1 level can be used to predict LV subclinical dysfunction in hypoxia-induced by PPHN with 3D STE parameters and the positive effects of melatonin on LV function.

Our study showed a negative correlation between serum NT-pro-BNP concentration and 2D LV GLS and GCS and 3D LVEF and 3D LV GLS in melatonin group, which means that its level can be used to predict LV subclinical dysfunction with 2D and 3D STE parameters.

On the contrary, Elmesiry et al. [13] who determined no substantial association between NT-pro-BNP levels and several 3D STE parameters of LV performance.

### Limitations

This research was performed at one center with a limited sample size, perhaps restricting the results’ generalizability. Advanced Echo and particularly for 3D Echo, where lower frame rates and the mismatch between neonatal body surface area and probe dimensions hindered data acquisition.

### Recommendations

A multicenter study with a larger sample size and a more diverse neonatal population would enhance the precision of conclusions regarding the use of melatonin as an adjunct in PPHN therapy to improve the outcome and protect the cardiac muscle from hypoxic injury. Also, future studies are needed to incorporate right ventricle ejection time to pulmonary artery acceleration time (RVET/PAAT) and shunt direction analysis for a more comprehensive evaluation of pulmonary hemodynamics.

## Conclusions

Melatonin can be used effectively as an adjunct along with the standard therapy for PPHN. It can also be used as a cardioprotective drug to protect the myocardial tissue from the effect of hypoxia induced by PPHN.

Research laboratory investigations were performed only after EOT, as transporting the hemodynamically unstable cases on a high-frequency oscillatory ventilator to the Echo laboratory was challenging at admission. Additionally, image quality posed a significant limitation for accurate stress and strain measurements.

## Supplementary Information

Below is the link to the electronic supplementary material.

Supplementary Material 1 (DOCX 20.1 KB)

## Data Availability

Data are available upon appropriate request.

## Abbreviations

BNP: B-type natriuretic peptide
cTDI: Color tissue Doppler imaging
EF: Ejection fraction
ELISA: Enzyme-linked immunosorbent assays
EOT: End of therapy
ESPAP: Estimated systolic pulmonary arterial pressure
FS: Fractional shortening
GCS: Global circumferential strain
GLS: Global longitudinal strain
HMGB1: High-mobility group box-1
LV: Left ventricle
MPA: Main pulmonary artery
MPI: Myocardial performance index
NICU: Neonatal Intensive Care Unit
NT-pro-BNP: N-terminal pro–B-type natriuretic peptide
PPHN: Persistent pulmonary hypertension of the newborn
PVR: Pulmonary vascular resistance
STE: Speckle-tracking echocardiography
TAPSE: Tricuspid annular plane systolic excursion
