# Supplementary material for: Melatonin effects on the left ventricular function in neonates with persistent pulmonary hypertension
Source: Eur J Pediatr. 2026 Apr 9;185(5):247. doi: 10.1007/s00431-026-06864-z (PMC13065569; doi:10.1007/s00431-026-06864-z)
Supplement: Supplementary file 1 — Supplementary Material 1 (DOCX 20.1 KB) [file 431_2026_6864_MOESM1_ESM.docx]

Table 1: Correlations between serum HMGB-1 concentrations after melatonin administration and Echo parameters

|  | | **Melatonin group** | | **Control group** | |
| --- | --- | --- | --- | --- | --- |
|  |  | **r** | **p** | **r** | **p** |
|  | **High mobility group box-1 concentration (pg/mL)** | | | | |
| **Conventional Echo after EOT** | ESPAP2 (mmHg) | –0.049 | 0.763 | –0.229 | 0.156 |
|  | MPA2 (mm) | –0.036 | 0.825 | –0.160 | 0.324 |
|  | LVEF2 (%) | 0.089 | 0.583 | 0.215 | 0.184 |
|  | LVFS2 (%) | 0.074 | 0.649 | –0.086 | 0.598 |
| **Tissue Doppler imaging** |  |  |  |  |  |
|  | LV free wall e'/a' | 0.306 | 0.055 | 0.211 | 0.191 |
|  | e' cm/s | –0.086 | 0.598 | –0.048 | 0.770 |
|  | a' cm/s | **–0.405** | **0.010*** | –0.309 | 0.052 |
|  | s' cm/s | 0.001 | 0.993 | –0.303 | 0.058 |
|  | LV MPI | **0.429** | **0.006*** | 0.283 | 0.077 |
| **2D STE** | LV-GLS (%) | **–0.529** | **<0.001*** | 0.291 | 0.068 |
|  | LV-GCS (%) | **–0.380** | **0.016*** | 0.171 | 0.290 |
| **3D Echo** | LVEF (%) | **–0.361** | **0.022*** | 0.263 | 0.101 |
|  | LVFS (%) | –0.043 | 0.790 | 0.123 | 0.451 |
|  | LV-GLS (%) | **–0.521** | **0.001*** | 0.242 | 0.133 |
|  | LV-GCS (%) | **–0.435** | **0.005*** | 0.294 | 0.066 |

r: Pearson coefficient, **p* ≤ 0.05. ESPAP: estimated systolic pulmonary artery pressure, MPA: main pulmonary artery, LVEF/LVFS: left ventricular (LV) ejection fraction/shortening, MPI: myocardial performance index, GLS/GCS: global longitudinal /circumferential strain.

Table 2: Correlations between serum NT-proBNP concentrations after melatonin administration and Echo parameters.

|  | | **Melatonin group** | | **Control group** | |
| --- | --- | --- | --- | --- | --- |
|  |  | **r** | **p** | **r** | **p** |
|  | **N-terminal pro B-type natriuretic peptide concentration (pg/mL)** | | | | |
| **Conventional Echo after EOT** | ESPAP2 (mmHg) | 0.066 | 0.684 | –0.076 | 0.640 |
|  | MPA2 (mm) | 0.034 | 0.836 | –0.030 | 0.855 |
|  | LVEF2 (%) | –0.085 | 0.602 | –0.012 | 0.942 |
|  | LVFS2 (%) | 0.105 | 0.520 | –0.032 | 0.846 |
| **Tissue Doppler imaging** | LV free wall e'/a' |  |  |  |  |
|  |  | 0.060 | 0.715 | –0.131 | 0.421 |
|  | e' cm/s | 0.030 | 0.852 | –0.197 | 0.222 |
|  | a' cm/s | 0.097 | 0.551 | –0.042 | 0.795 |
|  | s' cm/s | –0.007 | 0.966 | –0.094 | 0.565 |
|  | LV MPI | **0.463** | **0.003*** | 0.061 | 0.708 |
| **2D STE** | LV GLS (%) | **–0.357** | **0.024*** | 0.122 | 0.452 |
|  | LV GCS (%) | **–0.390** | **0.013*** | 0.230 | 0.154 |
| **3D Echo** | LVEF (%) | **–0.366** | **0.020*** | 0.131 | 0.422 |
|  | LVFS (%) | –0.055 | 0.737 | 0.005 | 0.975 |
|  | LV GLS (%) | **–0.378** | **0.016** | –0.170 | 0.293 |
|  | LV GCS (%) | –0.303 | 0.057 | –0.011 | 0.944 |

r: Pearson coefficient, ******p* ≤ 0.05. ESPAP: estimated systolic pulmonary artery pressure, MPA: main pulmonary artery, LVEF/LVFS: left ventricular ejection fraction/shortening, MPI: myocardial performance index, GLS/GCS: global longitudinal /circumferential strain.
